# The dog-leg design that can give clinical trials more power to their elbow

**DOI:** 10.1186/1745-6215-14-S1-P16

**Published:** 2013-11-29

**Authors:** Richard Hooper, Liam Bourke

**Affiliations:** 1 Queen Mary University of London, London, UK

## Background

A cross-over design is contraindicated in trials of interventions with curative or deep-rooted effects, which are not washed out in participants randomised to receive the control after the experimental intervention. A parallel groups design with or without a baseline assessment of outcome is commonly employed instead. We focus here on the situation where the control is routine care, or where the experimental intervention is the control plus something else, so that wash-out in participants moving from the control to the experimental arm is not an issue.

## Methods

We describe a simple but novel alternative design and its performance characteristics. One group is assessed before and after the experimental intervention, while two further, independent groups provide treatment comparisons at each time point. We call this a dog-leg design because of the pattern of assessments in the three groups.

## Results

If the correlation between baseline and follow-up is <0.72, the dog-leg design requires fewer participants than a parallel groups design with baseline assessment (i.e. an ANCOVA design) to achieve the same power. The dog-leg design also requires fewer assessments in total than a parallel groups design where participants are only assessed once, at follow-up.

## Conclusions

The dog-leg design is reminiscent of a stepped-wedge design, but with a reduced schedule of assessments, and with the notable difference that not all groups receive the intervention. There is a risk of differential attrition in the three arms, but the design's good performance relative to alternatives make it an attractive addition to the methodologist’s toolkit.

